# Prospective randomized evaluation of therapeutic decompressive craniectomy in severe traumatic brain injury with mass lesions (PRECIS): study protocol for a controlled trial

**DOI:** 10.1186/s12883-015-0524-9

**Published:** 2016-01-05

**Authors:** He-xiang Zhao, Yi Liao, Ding Xu, Qiang-ping Wang, Qi Gan, Chao You, Chao-hua Yang

**Affiliations:** Department of Neurosurgery, West China Hospital, Sichuan University, No. 37 Guoxue Xiang, Chengdu, Sichuan 610041 P. R. China; Department of Neuro-intensive care unit, West China Hospital, Sichuan University, No. 37 Guoxue Xiang, Chengdu, Sichuan 610041 P. R. China

**Keywords:** Decompressive craniectomy, Traumatic brain injury, Subdural mass lesions, Early surgery, Intracranial pressure

## Abstract

**Background:**

For cases of severe traumatic brain injury, during primary operation, neurosurgeons usually face a dilemma of whether or not to remove the bone flap after mass lesion evacuation. Decompressive craniectomy, which involves expansion of fixed cranial cavity, is used to treat intra-operative brain swelling and post-operative malignant intracranial hypertension. However, due to indefinite indication, the decision to perform this procedure heavily relies on personal experiences. In addition, decompressive craniectomy is associated with various complications, and the procedure lacks strong evidence of better outcomes. In the present study, we designed a prospective, randomized, controlled trial to clarify the effect of decompressive craniectomy in severe traumatic brain injury patients with mass lesions.

**Methods:**

PRECIS is a prospective, randomized, assessor-blind, single center clinical trial. In this trial, 336 patients with traumatic mass lesions will be randomly allocated to a therapeutic decompressive craniectomy group or a prophylactic decompressive craniectomy group. In the therapeutic decompressive craniectomy group, the bone flap will be removed or replaced depending on the emergence of brain swelling. In the prophylactic decompressive craniectomy group, the bone flap will be removed after mass lesion evacuation. A stepwise management of intracranial pressure will be provided according to the Brain Trauma Foundation guidelines. Salvage decompressive craniectomy will be performed for craniotomy patients once there is evidence of imaging deterioration and post-operative malignant intracranial hypertension. Participants will be assessed at 1, 6 and 12 months after randomization. The primary endpoint is favorable outcome according to the Extended Glasgow Outcome Score (5–8) at 12 months. The secondary endpoints include quality of life measured by EQ-5D, mortality, complications, intracranial pressure and cerebral perfusion pressure control and incidence of salvage craniectomy in craniotomy patients at each investigation time point.

**Discussion:**

This study will provide evidence to optimize primary decompressive craniectomy application and assess outcomes and risks for mass lesions in severe traumatic brain injury.

**Trial registration:**

ISRCTN20139421

## Background

Severe traumatic brain injury (STBI) is a major cause of death in young adults in developed countries [1]. In China, due to increasing traffic accidents, head injury occurs above 1 per 1000 [2]. The mortality of STBI is between 30 % and 50 %. According to a report from the Asian Development Bank, the annual economic loss from traffic accidents is equivalent to 1 %–3 % of China’s gross national product [3]. Considering the per capita income and limited medical resources, rational and effective treatment of STBI is of important economic and social significance.

Most STBI patients with intracranial hypertension attribute to mass lesions, such as contusion and subdural haematoma (SDH) [4, 5]. Severe primary injury and exasperate condition necessitate emergent surgical intervention. The available type of operation is either craniotomy or decompressive craniectomy (DC).

Craniotomy is the most widely used surgical treatment for STBI. This operation keeps the skull intact but the risk of post-operative refractory hypertension remains; thus, requires a salvage surgery. DC is another broadly used operation for STBI rescue, which involves the removal of the frontal, parietal and temporal skull to expand fixed cranial cavity. Some studies indicated that DC could effectively alleviate refractory intracranial hypertension [6–10]. However, using DC to treat STBI has been controversial since the 1970s [11, 12]. The focus of debate resides in whether DC would be able to improve the prognosis of patients. Current guidelines only suggest performing set the role of DC as a ‘surgical choice’ [5, 13].

Some studies which suggesting primary DC proposed that early aggressive intervention could mitigate the secondary damages of increased intracranial cerebral pressure (ICP) [10, 14–18]. In Europe, a retrospective study of 729 patients revealed that one-third of patients with STBI who received emergency surgery still needed DC even after haematoma evacuation [4]. Others who regarded DC as a second-tier salvage surgery considered that the long-term, frustrating recovery increased the uncertainty of DC [19]. Moreover, the complications remain perplex to surgeons [20, 21]. From the early stage of hemorrhagic progression to the late of cerebrospinal fluid disturbances, all these pathophysiologic changes would negatively impact the outcome of treatment [21–23]. Some authors pointed out that DC rests on the purpose of life-saving but costs severe disability in return [24, 25].

Except for fulminant intra-operative swelling, when surgeons take the risk of post-operative refractory hypertension into account, the decision of surgical option heavily relies on personal experiences. Thus, with the resurgence of DC, demands for compelling evidence regarding the use of DC in clinical practice have gathered momentum.

There are a few randomized controlled trials that have studied the use of DC for STBI. The results from completed trials cannot be generalized for solving the above questions [26]. DECRA study enrolled 155 cases of traumatic diffusion injury and the results suggested that DC did not show superiority over the best medical treatments [8]. Some commentators pointed out that the high crossover of patients from medical group to DC group in the DECRA could influence the outcomes [27]. Another notable trial is the RESCUE-ICP, which has collected data from 400 cases [28]. RESCUE-ICP permits craniotomy for mass lesion evacuation but excludes primary DC using. The results of this study remain to be concluded. RESCUE-ASDH, an ongoing trial, projects to compare primary DC and craniotomy in STBI patients with mass lesions [29].

However, it is common that the brain swells beyond the border of the skull window after mass lesion evacuation. Randomizing patients with brain swelling to craniotomy would impair patient's benefit. In contrast, screening out these patients would decrease study validity because intra-operative brain swelling is exactly indication for DC. In our hospital, primary DC is only selected for fulminant brain swelling, which we call therapeutic DC. The prophase study demonstrated that 54 % of patients reached a favorable outcome at 12 months after receiving therapeutic DC [30]. Even for some patients with fixed and dilated pupils, the bone flap could still be replaced. We also found there might be some predictive values of initial ICP for the risk of post-operative refractory intracranial hypertension [31]. All of these results need careful interpretation and prospective study confirmation.

Thus, considering the above background, we designed a clinical trial named prospective, randomized evaluation of therapeutic decompressive craniectomy in severe traumatic brain injury with mass lesions (PRECIS).

## Methods

### Study design

**Fig. 1:**
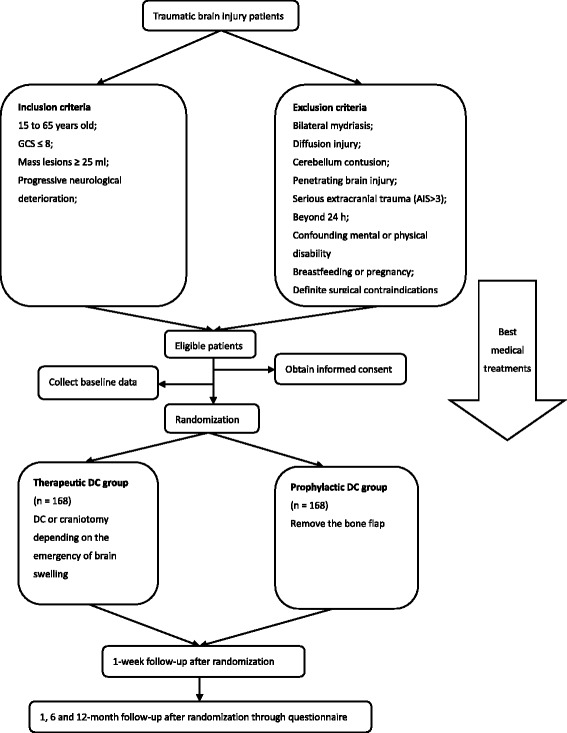
Flowchart of PRECIS outline

PRECIS is a prospective, randomized, assessor-blinded, controlled clinical trial designed at the West China Hospital, Sichuan University (Fig. 1). The study process will follow the Declaration of Helsinki [32]. The ethical approval was granted for the protocol version 1.2 by the Biological and Medical Ethics Committee (BMEC) of West China Hospital (No. 2015-17). The trial has been registered in the Current Controlled Trials (ISRCTN20139421). Whereas the neurologic status of STBI patients who will not be expected to give consent themselves, the lineal consanguinity or legal surrogate will be fully informed of the study purpose, procedure, potential risks and benefits. Before the trial process begins, a triplicate consent will be signed by a kin or a surrogate. Patients have the right to withdraw any time during the trial.

### The primary hypothesis

Therapeutic DC, which is performed on the basis of emergence of intra-operative brain swelling, results in a better outcome compared with prophylactic DC for STBI patients with mass lesions.

### The secondary hypothesis

1. Therapeutic DC results in fewer complications and a better quality of life compared with prophylactic DC
2. The risk of salvage craniectomy for post-operative refractory hypertension in primary craniotomy patients can be quantified by clinical features.

### Eligibility

#### Inclusion criteria

1. Between 15 and 65 years old;
2. Glasgow Coma Scale (GCS) ≤ 8;
3. Presence of high- or mix-density lesion ≥ 25 ml (contusion, intraparenchymal and SDH);
4. Progressive deterioration of neurological status after 24 h of injury (GCS motor score decreases by 2 points or blunt pupillary response);

#### Exclusion criteria

1. Bilateral mydriasis of critically endangered status;
2. Cerebellum contusion;
3. Penetrating brain injury;
4. Serious extracranial comorbidites with Abbreviated Injury Scale (AIS) > 3;
5. Beyond 24 h after injury;
6. Known mental or physical disability which would confound outcome assessment;
7. Breastfeeding or pregnancy;
8. Definite surgical contraindications;

### Interventions

To increase consistency between groups, most procedures are the same between the therapeutic DC group and the prophylactic DC group, except the bone flap management. Once the legal surrogate signs consent and confirms randomization, the intervention commences. Before the operation, the best medical treatments will be administered in accordance with the recommendation of the BTF guidelines [33]. For obtaining full course (initial, intra-operative and post-operative) of ICP and CPP variations, ICP monitor will be placed before the craniotomy and kept at least 5 days after surgery. Given the patients with mass lesion often associate with ventricles compression and midline shift, intraparenchymal ICP monitor will be preferred. The probe will be properly placed and fixed in the frontal lobe of lesion side drilling through the designed craniotomy incision without affecting normal procedure.

The unilateral trauma craniotomy model will be adopted, as suggested by Potts et al. [34]. The scalp flap should locate 1 cm beyond the border of the skull window. Craniotomy should reach at least 15 × 12 cm with fully exposed areas, which extended down to the temporal base and curve round the parietal to the side of the midline within 2 cm. After mass lesion evacuation, the dura should be sutured on relaxation and expansion by the temporal fascia or artificial dura. Then, for the prophylactic DC group, the bone flap will be removed. For the therapeutic DC group, the decision of whether or not to replace the bone flap will depend on brain swelling. If the brain tissue protrudes above the base of the temporal skull window, the bone flap will not be replaced.

General care monitoring is aimed to maintain SaO_2_ > 95 %, mean systolic blood pressure > 90 mmHg, temperature < 37 °C and PaCO_2_ 35-40 mmHg. Central venous catheters will be placed in all patients for volume monitoring and resuscitation.

As per the BTF guideline, a stepwise approach will be preferred to regulate ICP management. Maintaining ICP < 25 mmHg and CPP > 60 mmHg are the target of the critical care treatment. Conventional therapies include head elevation (30°), ventilation, sedation, osmotic dehydration, external ventricular drainage and neuromuscular blockade. The short active propofol (5–100 μg/kg/min) and fentanyl (25–100 μg/h) are used for sedation. Mannitol (1 g/kg) is routine diuretic for osmotherapy. Equiosmolar hypertonic saline (23.4 %, 0.686 ml/kg in bolus) will be considered in patients with refractory intracranial hypertension or rebound phenomenon treated by mannitol. The short half-life of vecuronium (0.2 mg/kg/h) is selected for neuromuscular paralysis agent. Hyperthermia (32 °C–34 °C) will be added to the STBI patients when ICP is refractory even maximal medical treatment.

During critical care, repeated computed tomography (CT) scan will assess intracranial evolution if ICP > 20 mmHg. For craniotomy patients, salvage DC will be performed when there are deterioration of imaging findings (midline shift > 5 mm and/or cisternal compression) and malignant intracranial hypertension (ICP 25–30 mmHg > 1 h or ICP > 30 mmHg, regardless time). For DC patients, even there is no consensus on the optimal time of cranioplasty [35], and systematic review also questioned the definition of “early” which varies from 1 to 4 months [36, 37], it is rational to recommend bone flap replacement as early as 2 months after surgery once their performances achieving the clinical standard.

Scheduled CT scan will be performed at 1, 3, 7, 30 and 180 days after surgery. Additional CT scan will carry out depending on the condition of patient.

### Follow-up and data collection

After randomization, patients will be assessed at 1 week and 1, 6 and 12 months. Patients who cannot be followed up at seven days will be assessed upon being discharged. For upfront exit patients, their surrogates should mark the previous data whether or not could be used in study. All patient information and imaging findings will be uploaded to a database using the case report form (CRF).

The initial data collection will be assessed at the emergency room by an attending physician. This collection will include baseline status, torso and/or extremities AIS, trauma severity (GCS, motor score and pupillary reaction), CT parameters (mass lesion type, volume of haematoma and midline-shift), time of injury-to-operation, cause of injury and initial laboratory features (arterial blood gas parameters, coagulation and platelet count).

The 1-week follow-up will include intra-operative brain swelling event, salvage craniectomy, laboratory features, early complications and ICP and CPP control. Intra-operative monitoring values will be recorded at the point of probe fixation, dura incision, dura suture and scalp closure. Post-operative ICP control will be recorded as daily ICP burden (number of hrs/d with ICP > 25 mmHg) and total ICP burden (number of days with ICP > 25 mmHg), as well as the same method recording daily and total CPP burden (CPP < 60 mmHg) [38].

At 1, 6 and 12 months follow-up, the independent blinded assessor will face-to-face evaluate intervention outcomes, quality of life, late complications and time of cranioplasty using standard questionnaire. The questionnaire includes the Extended Glasgow Outcome Score (GOSE), EQ-5D. If the discharged patients cannot attend scheduled follow-up, especially at time point of 6 or 12 months, the questionnaire will be sent to the documented permanent address. Only if these methods are invalid, the surrogate will be investigated to finish the questionnaire by telephone.

### Outcomes

The Primary endpoint is a favorable outcome at 12 months after randomization as measured by GOSE (5–8). The core secondary endpoint is the quality of life indicated by EQ-5D. Further secondary endpoints include mortality and complications at corresponding time point, ICP and CPP control and incidence of salvage DC in craniotomy patients.

### Statistical analysis

The analysis will be processed on the principle of intention. Patients who drop out or violate initial distribution will be considered as the worst outcome. Considering the probability of craniotomy patients treated by salvage DC, an as-treated analysis will also be implemented, as recommended in two-arm clinic trial [39]. Parametric data will be described by mean and standard deviation. Non-parametric data will be described by median and quartile. Categorical data will be presented using number and percentage.

The primary outcome of the study will be evaluated using the Pearson chi-square test with unadjusted risk ratio and 95 % confidence interval. Adjusted analysis of primary outcome will be assessed using logistic regression for pre-specified factors and any other covariates presenting significant difference between the two groups. The pre-specified subgroups include: age, GCS, pupillary response, mass lesion type and hypotension. Secondary outcome including assessment of quality of life, mortality at 6 and 12 months and incidence of complications will also be assessed using unadjusted analysis and adjusted regression. To investigate the risk factors of postoperative refractory hypertension (salvage DC), univariate and multivariate analysis will be used to identify potential variables of craniotomy patients.

Depending on the results of regression models, the receiver operating curves will be used to evaluate the discrimination. The Hosmer–Lemeshow test will process the goodness-of-fit of models [40]. A P value < 0.05 is considered statistically significant. All statistical analysis will be computed by SPSS software (version 19.0).

### Adverse events (AEs) and Serious adverse events (SAEs)

Both AEs and SAEs will be recorded in CRF. AE is defined as any unintended or unfavorable disease that occurs during the study. AEs include DC related complications, such as cortical herniation, hemorrhage evolution, infection, hydrocephalus and subdural hygroma. SAEs are defined as any of the following: death, life-threatening events, persistent vegetative state, requirement of hospitalization and prolonged hospitalization. Once the attending physicians realize SAE, it should be reported to the trial management team (TMT) and BMEC within 24 h. SAEs will be followed up until the issue is properly resolved.

### Sample size

The sample size calculation is on the basis of the variance of outcomes. The favorable outcome of DC in previous studies ranged from 30 % to 61 % [9, 15, 41–43]. Some results may not be applicable because they included patients with diffusion injury and used DC as a secondary therapy. When primary DC treated patients for mass lesions, the favorable outcome of prophylactic DC in 781 patients was 38 % [44–54]. In our centre using therapeutic DC, the favorable outcome of therapeutic DC was 54 % [30]. After including a safety margin of 10 % to account for patients who drop out, we calculate that a sample size of 336 (each arm 168) will be sufficient to reach a significance level of 5 % (two-side) and a power of 80 %. The sample size will be re-assessed and adjusted during interim analysis.

### Randomization

Computer-based central randomization will allocate treatment with minimizing following covariates: age (<40 or ≥ 40 years), GCS (8-6 or 5-3), pupillary response (both reactive or one reactive or no reactive), mass lesion type (subdural or intracerebral) and hypotension. A random element will be introduced to enhance the unpredictability of the minimization algorithm [55]. Physicians can use telephone or computer to register and randomize eligible patients. The patients will be assigned to either the therapeutic DC group or the prophylactic DC group and will receive a unique identity number for PRECIS records.

### Blinding

Since the study is conducted at a single center and interventions probably result in different outcomes and qualities of life, ‘ideal blinding’ is not realistic in PRECIS. We use an assessor-blinding method to investigate the study outcome. The independent assessors (IAs) will not be involved in the randomization or intervention. IAs will not be able to obtain the distribution information or the specific treatment procedures using CRF.

### Quality control and trial administration

#### Data safety monitoring board

The independent data safety monitoring board (DSMB) is established to ensure patient safety and data confidentiality. The members of DSMB include neurosurgeons, neurology physicians, ethicists and biological statisticians. The board members should have no conflicts of interests concerning the trial. DSMB is granted the highest authority to access the database. DSMB will launch interim analyses and further to recommend to the study group. The closed part of the interim analysis will be confidential to TMT and related staff. The recommendations will only suggest terminating the trial if one branch is significantly different (i.e. >3 SDs) with respect to primary endpoints or SAEs

#### Trial steering team

The trial steering team is responsible for supervising the overall process of the trial and final decisions following recommendations from DSMB.

#### Trial management team

The responsibilities of TMT include trial conception, intervention design, patient safety, quality assurance, data analysis and writing. TMT will periodically instruct each team to conduct coordination meetings. According to recommendations from the interim analysis, TMT will re-calculate and adjust the sample size if needed.

#### Trial executive team

As the main administrators of study implementation, veteran neurosurgeons constitute the trial executive team (TET). TET will conduct eligible patient screening, study interpretation, consent signature, AEs and SAEs reporting, and the CRFs filling. TET should also provide specific measures to improve the unqualified ratio and the timeliness of data submission following interim analysis.

#### Trial assistant team

The assistant team is composed of IAs and computer support group (CSG). Following the principle of blinding, IAs will complete study outcome assessment and submit to DSMB (owning priority) and TMT. CSG will perform the online random allocation system, database establishment and maintenance.

## Discussion

The bones of contention regarding the use of DC for patients with STBI are the treatment outcome and risk prediction. This should be settled through practical and objective clinical trials. PRECIS is the first, prospective, randomized trial to evaluate the outcome of therapeutic DC and prophylactic DC during the primary operation in STBI patients with mass lesions. Compared with completed trials and ongoing trials, PRECIS is relevant to the clinical practice and attentive to full course of core parameters variation. Excluding patients with intra-operative brain swelling could decrease the power of study, and designating these patients to have either craniotomy or DC would impair patient's benefit. We also try to identify our prior hypotheses about the risk of post-operative refractory intracranial hypertension. By focusing on general patients with mass lesions, this study wishes to describe the outcomes and prognosis of such patients following DC.

## Abbreviations

AE: Adverse event
BMEC: Biological and medical ethics committee
CPP: Cerebral perfusion pressure
CRF: Case report form
CSG: Computer support group
DC: Decompressive craniectomy
DSMB: Data safety monitoring board
GCS: Glasgow coma Scale
GOSE: Extended Glasgow Outcome Score
IA: Independent assessor
ICP: intracranial pressure
PRECIS: Prospective randomized evaluation of therapeutic decompressive craniectomy in severe traumatic brain injury with mass lesions
SAE: Serious adverse event
SDH: Subdural haematoma
STBI: Severe traumatic brain injury
TET: Trial executive team
TMT: Trial management team
